# Comparison of three different biomaterials used in in vitro molar apexification models

**DOI:** 10.1186/s12903-023-03180-y

**Published:** 2023-06-30

**Authors:** Elif Ece Kalaoglu, Canan Duman, Belen Sirinoglu Capan, Mert Ocak, Burak Bilecenoglu

**Affiliations:** 1 Faculty of Dentistry, Department of Pedodontics, Istanbul Gelisim University, Istanbul, Turkey; 2 Faculty of Dentistry, Department of Pedodontics, Istanbul Atlas University, Istanbul, Turkey; 3 Faculty of Dentistry, Department of Pedodontics, Istanbul University-Cerrahpaşa, Istanbul, Turkey; 4 Faculty of Dentistry, Department of Anatomy, Ankara University, Ankara, Turkey; 5 Faculty of Medicine, Department of Anatomy, Ankara Medipol University, Ankara, Turkey

**Keywords:** Apexification, Multi-rooted teeth, MTA, MTA Flow, Biodentine

## Abstract

**Objectives:**

New biomaterials had some advantages such as mixing and easier application as compared to traditional MTA in single step apexification method. This study aimed to compare the three biomaterials used in the apexification treatment of immature molar teeth in terms of the time spent, the quality of the canal filling and the number of x-rays taken to complete the process.

**Methods:**

The root canals of the extracted thirty molar teeth were shaped with rotary tools. To obtain the apexification model, ProTaper F3 was used retrograde. The teeth were randomly assigned into three groups based on the material used to seal the apex; Group 1: Pro Root MTA, Group 2: MTA Flow, Group 3: Biodentine. The amounts of the filling, the number of radiographs taken until treatment completion and the treatment duration were recorded. Then teeth were fixed for micro computed tomography imaging for quality evaluation of canal filling.

**Results:**

Biodentine was superior to the other filling materials according to time. MTA Flow provided greater filling volume than the other filling materials in the rank comparison for the mesiobuccal canals. MTA Flow had greater filling volume than ProRoot MTA in the palatinal/distal canals(*p* = 0.039). Biodentine had greater filling volume more than MTA Flow in the mesiolingual/distobuccal canals (*p* = 0.049).

**Conclusions:**

MTA Flow was found as a suitable biomaterial according to the treatment time and quality of root canal fillings.

## Introduction

The delayed diagnosis and treatment of deep carious lesions of immature permanent teeth can cause irreversible pulpitis, resulting in pulp necrosis. The damage may interrupt the root development process. The endodontic treatment of teeth with immature roots may be challenging due to their wide apices and fragile dentin walls. The treatment of this condition, which is frequently encountered by pediatric dentists in their clinical practices, is only possible with apexification procedures or revascularization treatment [1].

According to the definition of the American Association of Endodontics, apexification is a method that induces calcified barrier formation at the root tip of a permanent tooth with an open apex and necrotic pulpx [2]. The aim of the apexification procedure is to limit bacterial infection and create an environment that allows hard tissue formation in the apical region. The most common cause of endodontic treatment failure is the infiltration of irritants into periapical tissues. An ideal filling material should close the communication paths between the root canal system and the surrounding tissues In addition, it should not have toxic, genotoxic or cariogenic properties, and it should be biocompatible, insoluble in tissue fluids and dimensionally stable [3]. Many materials such as antiseptic or antibiotic pastes, tricalcium phosphate, camphor mono-chlorophenol and calcium hydroxide have been used previously to create an apical barrier. Apexification using calcium hydroxide as an intra-canal medication has been clinically accepted and has been the most widely used method for more than 40 years [4]. However, calcium hydroxide apexification has disadvantages such as prolonged treatment time, unpredictable apical closure, coronal micro leakage problems and difficulties in patient follow-up [5]. The prolonged use of calcium hydroxide weakens the root structure by dissolving, denaturing and neutralizing the acidic components of dentin [6].

In order to overcome these disadvantages, a single-step apexification method using mineral trioxide aggregate (MTA) was proposed as an alternative method in 1993 [7, 8]. It has been shown that MTA is a material with high biocompatibility, suitable for inducing the formation of an apical hard tissue barrier in necrotic pulped teeth with incomplete root development. There are many factors that contribute to the increase in popularity of this material [9]. Besides being nontoxic, it stimulates cementogenesis. Moreover, it creates a highly alkaline environment by releasing calcium and hydroxyl ions [10]. Numerous studies have shown that MTA is radiopaque and antimicrobial. It also has high dissolution resistance and stimulates odontoblast differentiation. However, the long curing time and the difficulty of application have led to the search for new biomaterials, especially for the treatment of multi-rooted teeth [7]. Although, there are many studies showing that MTA apexification successfully provides apical closure, there are no studies with multi-rooted teeth showing the adaptation of MTA to the root canals and canal filling quality.

MTA Flow™ is a calcium silicate-based material with a smaller particle size and a purer composition as compared to conventional MTA. The particle size of the powder is less than 10 microns (µm). Its other physical properties are the same as those of the conventional MTA. In addition, the curing time has been reduced to 15 min. Unlike the conventional MTA, the mixture formed when the powder and liquid are mixed does not have a sand-like consistency, which facilitates the application of the material [11].

Biodentine™ is a material that contains tricalcium silicate and dicalcium silicate, and which bioactively mimics dentin in the crown or the root of the tooth using active biosilicate technology [12]. Its 12-min hardening time and high mechanical resistance that allow for easier application makes it superior to MTA [11, 13]. Following the hardening process, its pH is 11.7, and its particle size is 5 µm. Its mechanical properties are superior to those of MTA, and it is more resistant to acids [12].

There is no study in the literature that evaluates the three materials described above in terms of ease of application and filling quality in multi-rooted teeth. Current publications on the treatment of molar teeth, which have many clinical difficulties in terms of application in the treatment of apexification, are limited to case reports.

The aim of this study is to compare the three materials used in the apexification treatment of immature molar teeth in terms of the time spent, and the number of x-rays taken to complete the process. In addition, the quality of the canal filling was evaluated using Micro CT.

## Materials and methods

### The universe and sample size of the study

The Dean’s Office of Biruni University Faculty of Dentistry at Istanbul, Turkey allowed this study to be carried out in university clinics. The study was conducted in accordance with Declaration of Helsinki and was approved by Biruni University Non-Interventional Clinical Research Ethical Committee (Approval No. 2019/25–02). In our study, teeth extracted at our faculty between March and August 2019 were included. Before the tooth extraction, each patient signed the consent form for the use of extracted teeth in any study which will be conducted in Biruni University.

The procurement of the extracted teeth, the apexification applications and the x-rays were carried out at Biruni University Faculty of Dentistry, Istanbul, Turkey. Micro-CT analyses of the applied teeth were carried out at Ankara University Faculty of Dentistry, Ankara, Turkey.

In the literature, similar studies that were previously performed were examined when determining the sample volume. When determining the effect size to be used in power analysis, the article entitled “Evaluation of Root Canal Sealer Filling Quality Using a Single-Cone Technique in Oval Shaped Canals: An in vitro Micro-CT Study” and the G POWER 3.1 package program was used [14]. As a result of the calculations, the minimum sample size to provide the power of the test (1-β) = 0.80 was determined as 30 (10 in each group) in total.

### Inclusion and exclusion criteria

Extracted permanent molar teeth with complete root development and without root resorption and root fracture were included in our research.

Teeth with external root resorption, root fracture, and whose root canals were calcified in a way that did not allow access to the apical were excluded.

### Clinical procedure

In this study, 30 molar teeth, that were extracted for reasons independent of our study (non-restorative caries, periodontal damage, and periapical lesion), were included. Soft tissue, calculus, and bone residues from root surfaces were removed with a Gracey curette. After this process, the teeth were kept in 5.25% sodium hypochlorite for 2 h to ensure root surface disinfection and then were kept in distilled water until the start of treatment. The specimens were decoronated from the enamel-cement boundary under water cooling with a diamond disc. The length of each root was adjusted to be 10 mm. The working length was determined and recorded visually by advancing stainless steel files into the root canal apically until it was visible. To obtain the apexification model with an open apex, ProTaper F3 (Dentsply, Maillefer, Ballaigues, Switzerland) was used as retrograde instrumentation. The root canals of the teeth, which were held with moist gas pads to prevent drying, were shaped with rotary tools to have a master cone size of 45 by a single researcher (BSC). After each file, canal irrigation was provided with 1 ml of 2.25% sodium hypochlorite. The final irrigation was performed with 2 ml of 17% EDTA, followed by 2 ml of 5.25% sodium hypochlorite using the traditional method. Then the root canals were dried with paper points. The samples were randomly numbered and divided into three groups by the same researcher (BSC) to be embedded in paraffin with the apical half of the roots closed. All root fillings in the three groups were performed by another experienced researcher (EEK). In the preparation of all three materials, the mixing ratios and instructions of the relevant manufacturers were followed for apexification.

Group 1: ProRoot MTA Manual Carrier (Dentsply Sirona) was used to carry MTA into the canals. The apical parts of the roots were filled with mineral trioxide aggregate (MTA; ProRoot; Dentsply Tulsa Dental) by using paper points and condensed using an endodontic plugger (Fanta Dental, 59 GP Plugger, #4, Niti Head[#60/03], SS Head[#120/029]).

Group 2: The apical parts of the roots were filled with MTA Flow (Ultradent Products Inc., South Jordan, UT, USA, lot: 2,015,122,901) by using special applicators (Skini syringe and NaviTip™ Tip [29 ga]). After mixing MTA flow, it was inserted into the clear Skini delivery syringe and used recommended NaviTip™ Tip [29 ga]. The tip was placed 1–2 mm short from the apical stop and MTA Flow was gently delivered into the canal. Vertical compressing forces during obturation were avoided.

Group 3: Biodentine (Septodont Inc., Saint-Maur-des-Fossés, France, lot: B18542A) was collected by the instrument supplied in the box and moved into the canal by a spatula. The apical parts of the roots were filled with Biodentine by using paper points and condensed using an endodontic plugger (Fanta Dental, 59 GP Plugger, #4, Niti Head[#60/03], SS Head[#120/029]).

During these apical plug condensation, periapical x-rays were taken to confirm placement of the biomaterials in the last apical 3–4 mm of the canal length. If an adequate barrier was not created, re-preparation and/or condensation of the biomaterials was continued.

Digital periapical radiographs were taken for all teeth in all three groups by an experienced radiology technician using a digital phosphor plate radiography system (Dentsply Sirona Vario DG, Assago-Milano, Italy) using the parallel technique for standardization, respectively. One pediatric dentist who was blinded to the study group (CD) observed and evaluated the radiographs using the Picture Archiving and Communication Systems software version (1.1.1.6) for Windows 10 (Microsoft Corporation, Redmont, WA, USA) and displayed on a 28-inch Samsung LU28H750UQMXUF monitor (Samsung Electronics, Seoul, South Korea) with a 3,840 × 2,160-pixel resolution. The same blinded researcher recorded the number of radiographs taken until treatment completion and the treatment duration. Periapical x-rays were taken until the researcher (CD) saw that an apical barrier of at least 4 mm was formed. After x-ray confirmation of an adequate filling in the apical region, cotton pellet moistened with saline placed over ProRoot MTA for 24 h. In MTA Flow and Biodentine groups, placing wet gauze was not applied by following the manufacturers’ instructions. After the condensation of the biomaterials, the canals were sealed with temporary filling (Cavit, 3 M ESPE, St Paul, MN, USA) and kept at room temperature for one week prior to micro-CT imaging.

### Micro computed tomographic evaluation

**Fig. 1:**
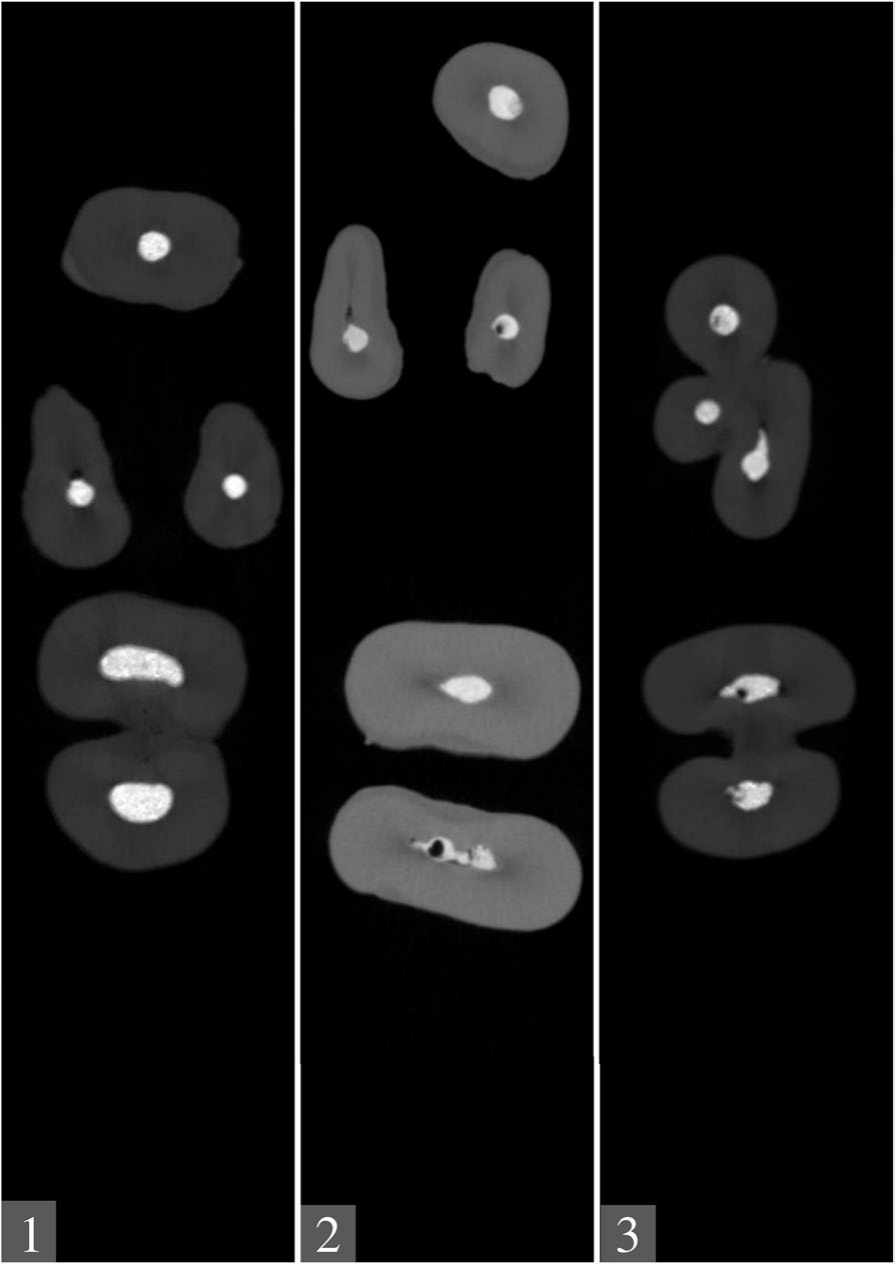
Micro-CT images of Group 1; ProRoot MTA, Group 2; MTA Flow, Group 3; Biodentine

Teeth with apical filling were fixed for micro computed tomography (CT) imaging (Fig. 1). All images were taken with a high-resolution micro CT device (Skyscan 1275, Kontich, Belgium) at Ankara University Faculty of Dentistry. The images were acquired by a single researcher (BB) using 100 kV, 100 mA, and a 0.5-mm-thick Al/Cu filter, with 360° rotations on the vertical axis and the acquisition parameters. The images were evaluated with NRecon 1.6.9.4 SkyScan 2011 and DataViewer 1.5.0 64-bit software program. Each root canal was divided into three regions as: 0–3 mm (apical), 3–6 mm (middle), and 6–10 mm (coronal) for the evaluation of voids and filling.

Although the root canal lengths are standardized at 10 mm, the canal diameter of each tooth of each canal is different. It is therefore not possible to keep the volumes same. The mean percentages of the canal filling material volume (sum of the volume of the biomaterial), the volume of internal voids (unfilled spaces in the biomaterial), the external voids (spaces along the canal walls), and the combined voids were calculated by another blinded researcher (MO) using micro-CT analysis. The volume of the canal filling material for each canal was calculated by subtracting the volume of the voids from the total volume of the apical region. The ratio of the filling volume to the volume of the total apical region was calculated as a percentage and comparisons were made as a percentage of filling to avoid bias.

Root canals were grouped according to their mean diameters for material comparison. As the order of the canal diameters is palatinal (P) > mesiobuccal (MB) > distobuccal (DB) in the upper teeth and distal (D) > mesiobuccal (MB) > mesiolingual(ML) in the lower teeth, the canals were evaluated by dividing them into three groups: P/D, MB, and ML/DB.

### Statistical analysis

Data were analyzed using the SPSS version 25.0 statistical software (IBM). Two-tailed *p* values < 0.05 were considered statistically significant. As the variables used in our study did not have a normal distribution and our sample size was < 30, we compared the samples with each other using the Wilcoxon test and a triple analysis with the Friedman analysis of variance (ANOVA). Three materials were compared with each other using the Friedman ANOVA. As a triple comparison was made, the *p* value was calculated with Bonferroni correction.

## Results

The mean filling times of the three materials were compared. As shown in Table 1, the filling time was fastest with Biodentine and slowest with ProRoot MTA. When the number of periapical x-rays taken was evaluated, 2 x-rays were taken at the rate of 50% for ProRoot MTA (1 x-ray:50%), 3 x-rays at the rate of 60% for MTA Flow (1 x-ray:20%,2 x-ray: %10, 4 x-ray:10%) and 2 x-rays at the rate of 60% for Biodentine (1 x-ray:10%, 3 x-ray:30%). It was determined that the material for taking the least number of periapical x-rays was the ProRoot MTA. Tables 2, 3, and 4 show the mean values, standard deviations, minimum, maximum values, and median values for the MB, ML/DB, and P/D material volumes, respectively. In the P/D canals, the filling rates of ProRoot MTA and Biodentine were similar, whereas MTA Flow had a slightly higher filling rate (Table 2). When we examined the filling rates in the MB canals, we found that ProRoot MTA and Biodentine had similar filling rates, whereas MTA Flow had a higher filling rate (Table 3). We determined that the difference in filling volume between the materials was mostly in the ML/DB canals, which are the narrowest canals. It was observed that ProRoot MTA provided less filling than MTA Flow and Biodentine. The MTA Flow provided the highest filling rate (Table 4). The Friedman ANOVA revealed significant differences in time and MB volume in the triple comparisons but no significant differences in the comparisons of the other two volumes. A significance of *p* = 0.002 < 0.05 was found over time; the p value was calculated as 3 × 0.002 = 0.006, with Bonferroni correction because three variables were compared. Biodentine was superior to the other filling materials in the rank comparison according to time. MB volume had a significance of *p* = 0.006 < 0.05, calculated as 3 × 0.002 = 0.018, with Bonferroni correction because three variables were compared. MTA Flow provided greater filling volume than the other filling materials in the rank comparison for the MB canals. The comparisons of the groups in pairs using the Wilcoxon test were examined. The comparisons of time and MB, ML/DB, and P/D material volumes for all three materials revealed that some of the materials significantly differed in time, P/D, ML/DB, and MB material volumes. According to time, the significance of Biodentine compared with that of ProRoot MTA was very high (*p* = 0.009). MTA Flow had greater filling volume than ProRoot MTA in the P/D canals (*p* = 0.039; *p* = 0.015 < 0.05 had significance after Bonferroni correction of material 2 according to material 1). Biodentine had greater filling volume than MTA Flow in the ML/DB canals (*p* = 0.049). Tables 1, 2, 3, and 4 present the descriptive statistical values of the study, namely mean, standard deviation, minimum, maximum, and median values. The values were close to each other, except for the mean and standard deviation of time. According to these data, MTA Flow was predicted to be the most useful material, considering its highest rank ratio and the pairwise comparisons of the materials.

**Table 1:** Descriptive statistics for time

	**N**	**Mean**	**Std. Deviation**	**Minimum**	**Maximum**	**Percentiles**		
						**25**^**th**^	**50th (Median)**	**75th**
**ProRoot MTA**	10	729,0500	194,17539	450,50	1021,00	577,1250	708,0000	919,7500
**MTA Flow**	10	492,8560	115,21278	374,33	766,00	425,2475	443,7050	553,5400
**Biodentine**	10	465,6490	140,48306	322,50	797,50	353,8300	438,1650	532,1250

**Table 2:** Descriptive statistics for the Volume of PALATINAL/DISTAL Canals

	**N**	**Mean**	**Std. Deviation**	**Minimum**	**Maximum**	**Percentiles**		
						**25**^**th**^	**50th (Median)**	**75th**
**ProRoot MTA**	10	,89989	,02158000	,86811821	,938212580	,88470718500	,89737927000	,9135988950
**MTA Flow**	10	,92095	,01550465	,89389200	,948045750	,91338416000	,91867698500	,9324354850
**Biodentine**	10	,89433	,01654298	,87242962	,921053931	,87951915950	,89561020750	,9047031405

**Table 3:** Descriptive statistics for the Volume of MESIOBUCCAL Canals

	**N**	**Mean**	**Std. Deviation**	**Minimum**	**Maximum**	**Percentiles**		
						**25**^**th**^	**50th (Median)**	**75**^**th**^
**ProRoot MTA**	10	,910886	,009391850	,8966345	,9261674	,903094455	,908729255	,919143580
**MTA Flow**	10	,928412	,010147710	,9137685	,9433558	,917905065	,928144060	,937400522
**Biodentine**	10	,910391	,024167360	,8496026	,9307109	,903833539	,912458758	,928803721

**Table 4:** Descriptive statistics for the Volume of MESIOLINGUAL/DISTIBUCCAL Canals

	**N**	**Mean**	**Std. Deviation**	**Minimum**	**Maximum**	**Percentiles**		
						**25**^**th**^	**50th (Median)**	**75th**
**ProRoot MTA**	10	,82322	,2895205831	,0000000	,9433699	,903416,988	,90961545	,9210155
**MTA Flow**	10	,92723	,0083970116	,9147169	,9398844	,920626095	,92699781	,9349848
**Biodentine**	10	,90662	,021704798	,870178	,927951	,88449049	,91581162	,9229667

## Discussion

The traditional apexification technique is a long-term calcium-hydroxide therapy and has some disadvantages such as long treatment time, follow-up difficulties, unpredictable apical closure, root fractures, and delayed treatment [15]. An alternative technique is to make an apical plug with a biocompatible material [16]. Root canal treatments are difficult to perform in pediatric patients. Apexification is more difficult to perform in multi-rooted teeth because accessing the apex and making an apical plug in the right location is more challenging. For these reasons, in clinical practice, the material for apexification must be easy and fast to use (preparing and filling) to provide sufficient apical closure. ProRoot MTA was the first commercially available and most researched MTA cement [17]. It uses the traditional powder-water mixing technique and has poor handling characteristics [18]. In clinical practice, it is not easy to apply owing to its sand-like mixture. Biodentine and MTA Flow had both good physical and biological properties. Their application techniques and procedures are easier than those of the conventional MTA [15].

Natural human molar teeth were used in this study because we tried to simulate real clinical conditions and wanted to see how the difficulties of root canal morphologies would be reflected in the study results.

Most apexification studies were performed in single-rooted teeth [9, 10, 19]. A study compared the marginal adaptation of calcium silicate-based root filling materials and used only palatal roots of the maxillary molars [20]. A micro-CT study was performed to assess the porosity distribution of Bio Root RCS and MTA Flow in mandibular molars. However, only the mesial canals were evaluated [21]. Although both studies were performed on multi-rooted teeth, other canals were not included in the study, and no comparison was made among the canals. The aim of our study was to select the appropriate dental material for successfully filling narrower and curved canals that requires the least number of radiographies and offers the best apical closure, which is one of the most clinically challenging treatments. In the present study filling volumes were analyzed separately in mesial, distobuccal/lingual, and palatinal/distal canals for each filling material. The filling rates of the materials significantly differed among the canals. We found that MTA Flow provided greater material volume in the mesiolingual/distobuccal canals. This may be due to the consistency of the product and its special syringe, which make it easy to apply and access narrow canals. Microleakage and sealing ability studies between traditional MTA and Biodentine used single-rooted teeth [22–24]. Kokate et al. conducted a study in which MTA, glass ionomer cement, and Biodentine were evaluated in terms of microleakage by dye penetration and found the least microleakage with Biodentine [24]. Refaei et al. compared the sealing efficiencies of Pro Root MTA, Biodentine, and a calcium-enriched mixture in open-apex teeth [23]. By using the bacterial leakage method, they found that Biodentine was a better sealer than Pro Root MTA. Tang et al. also showed that Biodentine was a significantly superior sealer than MTA in root-end filling [22]. In our study, Biodentine and Pro Root MTA had similar filling rates, but Biodentine had slightly higher filling rates than Pro Root MTA in the ML/DB canals.

This study also evaluated the treatment durations. Treatment duration might become the most important criteria for pediatric dentists in patients with whom cooperation cannot be achieved and inappropriate for general anesthesia/sedation. Our results indicated that Biodentine was superior to MTA Flow and Pro Root MTA in terms of filling speed, which is in accordance with the findings of previous studies [15]. MTA Flow was found to be more successful than Biodentine and Pro Root MTA in apexification in ourstudy. In our literature review, we found no study that compared the filling rates of these three materials in the open apex. MTA Flow is a novel material with better handling and manipulation properties than traditional MTA. Published studies focused on the cytotoxic effects, chemical and physical properties, biocompatibility and biomineralization, and radio-opacity of MTA Flow [25–27]. An in vivo animal study claimed that inflammation responses did not significantly differ between MTA Flow, ProRoot MTA, and MTA Angelus after 30 and 60 days [28]. Another study found that MTA Flow and ProRoot MTA had similar cytotoxic effects on human gingival fibroblasts [29]. In light of these studies, we wanted to examine MTA Flow in an apexification study and compare filling speeds and amounts that had not been investigated before. When all the canal filling rates were evaluated, MTA Flow was found to have the highest filling rates. When speed was added to the evaluation criteria, MTA Flow was again found to be the most successful and suitable material for apexification. As the root canal diameters and volumes of all molar teeth used in the study were different, the filled and void volumes were calculated in cubic millimeter ratios in the apical tip and converted into filling volume percentages in accordance with other micro CT studies [30]. None of the biocompatible materials in our study could completely fill the 3-mm apical region. Drukteinis et al. also claimed that void-free root fillings in the apical regions of the curved roots of the mandibular molars could not be achieved using BioRoot or MTA Flow [21].

The limitation of our study was that it was an in vitro study, although in vivo environmental conditions were applied (clinical setting and periapical radiography). Therefore, root canal filling procedure was easier than in vivo practice. This may have increased the success percentages for each material. Additionally, operating clinician’s experience in using a particular material may affect the canal filling time. To avoid bias, all root canal shaping, root canal fillings, and measurements were performed blindly by different researchers.

This study is the first study in the literature which compared the three biomaterials (ProRoot MTA, Biodentine and MTA Flow) used in the apexification treatment of immature molar teeth in terms of the time spent, the quality of the canal filling and the number of x-rays taken to complete the process. This is also the first study in literature which conducted in multi-rooted teeth comparing the biomaterial volumes among canals.

Our study provides enlightenment and guidance by conducting an in vitro apexification study to compare the filling success rates of different filling materials for the root canals of the teeth. The filling success rates of continuously developing dental biomaterials in clinical practice were compared. Further studies are needed in the field of apexification in multi-rooted teeth.

## Conclusions

Apical fillings were completed in a shorter time with Biodentine than other materials, while MTA Flow was found to be useful in terms of consistency of material and filling adequately in curved canals. For pediatric dentistry, in addition to the filling volume at the apex, the ease of use of the material, the duration of treatment and the number of periapical X-rays is important. When all root canals and these parameters were evaluated, MTA Flow was found to be the most suitable apexification material that provides the highest filling rate in the shortest time.

## Data Availability

All data generated or analyzed during this study are included in this published article. The raw data is available from corresponding author on reasonable request.
